# A Pilot Study to Assess At-Home Speed of Processing Training for Individuals with Multiple Sclerosis

**DOI:** 10.1155/2019/3584259

**Published:** 2019-06-03

**Authors:** Lindsay Barker, Brian C. Healy, Emily Chan, Kaitlynne Leclaire, Bonnie I. Glanz

**Affiliations:** ^1^Partners Multiple Sclerosis Center, Brigham and Women's Hospital, Boston, MA, USA; ^2^Department of Neurology, Harvard Medical School, Boston, MA, USA; ^3^Biostatistics Center, Massachusetts General Hospital, Boston, MA, USA

## Abstract

**Objective:**

Cognitive impairment is a common symptom of multiple sclerosis (MS), yet treatment is currently limited. The primary goal of this pilot study was to assess the feasibility and acceptability of an at-home, five-week computerized speed of processing (SOP) training intervention for MS patients. In addition, we examined the utility of the intervention to improve speed of information processing, memory, executive function, and health-related quality of life (HRQOL).

**Method:**

Fifteen subjects were assigned five weeks of SOP training, two times per week, for a total of ten sessions. Subjects were trained on five computerized SOP tasks that required processing of increasingly complex visual stimuli in successively shorter presentation times. Subjects were given a neuropsychological test battery that included measures of speed of information processing, verbal memory, visual spatial memory, and executive function. Subjects were also administered patient-reported outcome (PRO) measures to assess HRQOL, depression, and work productivity. Neuropsychological and PRO batteries were completed at baseline and after five weeks.

**Results:**

Eighty percent of subjects completed the five-week intervention (n = 12). Significant improvements were observed on some, but not all, measures of speed of information processing, verbal memory, and executive function. There were no significant changes in HRQOL.

**Conclusion:**

This pilot study supports the feasibility of an at-home SOP training intervention for individuals with MS. SOP training was associated with improvements in several cognitive domains. Larger, randomized controlled trials are warranted.

## 1. Introduction

Cognitive impairment is a key feature of multiple sclerosis (MS), affecting between 40 and 65% of patients at some point in the course of the disease [1, 2]. Deficits commonly involve speed of information processing, attention, verbal and visual spatial memory, and executive function [3]. Cognitive dysfunction can occur early in the disease process [4, 5] and decline may be seen over periods ranging from one to 10 years [6, 7]. Cognitive impairment is associated with significant patient morbidity, impacting employment [8, 9], social functioning [10], treatment adherence [11], and health-related quality of life (HRQOL) [12, 13]. The treatment of cognitive dysfunction in patients with MS has proved to be challenging. Standard disease modifying therapies have shown weak positive effects on cognitive function in MS with various methodological issues limiting any firm conclusions [14–16]. Additionally, no effective symptomatic treatments have been identified [17–20].

Cognitive remediation is a behavioral intervention consisting of training activities aimed at improving cognitive function. Reduced speed of information processing is a core deficit in MS [21] and it may impact related cognitive abilities such as learning and memory and executive function. A number of studies in healthy older adults have demonstrated improvement in processing speed and everyday functional activity following speed of processing (SOP) training using a program developed by Ball et al. [22] and tested in the Advanced Cognitive Trial for Independent and Vital Elderly (ACTIVE) [23]. Eighty-seven percent of ACTIVE subjects who underwent SOP training demonstrated improvements in processing speed that were maintained at five [24] and ten years [25]. Additionally, a recent follow-up to the ACTIVE study found a 29% reduction in the risk of dementia for the SOP training group after 10 years of follow-up compared to controls [26]. In addition to older adults, SOP interventions have been successfully applied in patients with HIV [27] and breast cancer [28].

The effect of SOP training on cognitive function in MS has been investigated in several small studies which found improvements in cognitive function using combined SOP and working memory interventions [29–31]. Some promising support for cognitive remediation in MS to date comes from a recently published randomized controlled trial [32]. Following 6 weeks of at-home training, the cognitive remediation group showed greater improvements on a neuropsychological composite measure relative to the active control group. However, this study utilized a cognitive training program targeting multiple cognitive functions including processing speed, attention, working memory, and executive function. The investigation of SOP remediation on cognitive function in MS has been absent from the literature, which is surprising given that SOP is considered a key deficit that likely underlies other cognitive functions.

The primary goal of this pilot study was to assess the feasibility and acceptability of a five-week, at-home SOP training intervention for MS patients. In addition, we examined the utility of the SOP training intervention to improve speed of information processing, memory, executive function, self-reported functional activities, emotional function, and HRQOL.

## 2. Materials and Methods

### 2.1. Subjects

Subjects were recruited from the Comprehensive Longitudinal Investigation of Multiple Sclerosis at the Brigham and Women's Hospital, Partners MS Center (CLIMB). The CLIMB is an ongoing prospective observational cohort study that began enrolling subjects in 2000 [33]. Subjects were approached about study participation at the time of their annual CLIMB visit by their treating neurologist. Inclusion criteria included (1) clinically definite MS according to the revised McDonald criteria [34], (2) age 18-60, (3) no history of learning disability, (4) no history of major depression, (5) no history of drug or alcohol abuse, (6) no history of neurologic disorder or head injury other than MS, (7) no history of severe visual loss, (8) no exacerbation in the past 30 days, (9) no steroid use in the past 30 days, and (10) English language skills adequate for cognitive testing and the completion of questionnaires. Interested individuals met with a member of the study staff to learn about the study in more detail. Fifteen subjects were enrolled. Subjects had a mean age of 47 +/- 6.3 years and a mean disease duration of 14.4 +/- 5.6 years. Additional demographic and baseline clinical characteristics of the study subjects are provided in Table 1. This study was approved by the Partners Human Research Committee at the Brigham and Women's Hospital and all subjects provided written, informed consent.

### 2.2. Measures

Prior to SOP training, subjects were administered the Brief Repeatable Battery of Neuropsychological Tests in Multiple Sclerosis (BRB) [35]. The battery includes the Symbol Digit Modalities Test (SDMT) (speed of information processing), Paced Auditory Serial Addition Test (PASAT) (speed of information processing), Selective Reminding Test (SRT) (verbal memory), 10/36 Spatial Recall Test (10/36) (visual spatial memory), and Controlled Oral Word Association Test (COWAT) (verbal fluency and executive function). Subjects were also administered the Stroop Test, an additional measure of speed of information processing and executive function [36]. Finally, they were given the following patient-reported outcome (PRO) measures: Medical Outcomes Study Short Form 36 Health Survey (SF-36) [37], Center for Epidemiologic Studies Depression Scale (CES-D) [38], and Work Productivity and Activity Impairment Questionnaire (WPAI) [39]. Within one week of completing SOP training, subjects were regiven the BRB, Stroop Test, SF-36, CES-D, and WPAI. Alternate versions of the verbal and visual spatial memory tests were used. Cognitive and PRO measures were administered by a research assistant (EC, KL) under the supervision of a neuropsychologist (LB).

### 2.3. Intervention

Subjects completed five weeks of at-home, computerized SOP training developed by Posit Science. Sessions were 45-60 minutes long and were completed two times per week, for a total of 10 sessions. The first training session was completed at the Partners MS Center. Each subject was provided with a unique email address and password to access the training website (http://www.brainhq.com) from home to complete additional training sessions using a desktop computer, laptop, or tablet. Study staff checked in with subjects once per week to troubleshoot any issues and assess compliance. Subjects were also given contact information to reach study staff in case they had any questions or problems.

The training package (Visual Rehabilitation, Posit Science) was originally developed as part of the ACTIVE [22] trial and then refined over time [27, 28, 40]. Subjects trained on five computerized SOP tasks involving target detection, identification, discrimination, and localization: Double Decision (formerly known as Road Tour), Target Tracker, Hawk Eye, Visual Sweeps, and Eye for Detail. Although the tasks varied slightly in stimuli presentation and requirements, they shared the same visually based speed component that required subjects to process increasingly complex visual stimuli in successively shorter presentation times. Task difficulty was automatically adjusted to user performance to maintain an 85% correct rate. The presentation of SOP tasks was designed such that 60% of time was spent on Double Decision, the primary SOP task used in the ACTIVE trial.

### 2.4. Statistical Analysis

The proportion of subjects who completed the intervention was calculated along with the exact binomial 95% confidence interval. Summary statistics for each of the cognitive tests and PROs were calculated for all subjects and in the subset of subjects who completed the intervention. To estimate the effect of the training on each of the outcome measures (cognitive and PRO), we fit a linear mixed effects model with a categorical effect of time and a random intercept. We fit this model in all subjects including those who only had a baseline measurement as the primary analysis. We also fit the model only in the completers. All statistical analyses were completed in the statistical package R (www.r-project.org/).

## 3. Results

Of the 15 subjects who enrolled, 12 completed the intervention (proportion completed=0.8; 95% CI: 0.52, 0.96). In terms of completed sessions, seven out of the twelve subjects who completed the intervention completed all 10 training sessions (proportion=0.58; 95% CI: 0.28, 0.85), four subjects missed one out of 10 sessions, and one subject missed two sessions. Of the subjects who withdrew, one cited difficulties with her peripheral vision that interfered with the visual tasks. Two other subjects withdrew for unclear reasons.

The summary statistics for cognitive measures and the estimated change in each of these measures after the intervention are provided in Table 2. The results show significant improvement in several cognitive domains analyzing either data from all subjects or just completers. When all subjects were analyzed, significant improvements were observed in verbal memory (SRT Long Term Storage, p<.01, and Consistent Long Term Retrieval, p<.01) and some measures of processing speed (2-second trial of the PASAT, p<.01, Stroop Word, p<.05). When only completers were analyzed, the results were similar with the exception that significant improvement was also observed on a measure of executive function (Stroop Color-Word Trial, p<.05). Further, subjects showed improvement across all other cognitive domains, although these changes did not reach significance. No significant changes in PROs were observed (see Table 3).

## 4. Discussion

The primary goal of this study was to assess the feasibility and acceptability of an at-home, five-week SOP training intervention for MS patients. Eighty percent of subjects completed the five-week intervention (n = 12), and, of those, 58% completed all 10 training sessions (n = 7). These compliance rates are comparable to other studies using remote cognitive interventions [30, 32] and support the idea that at-home cognitive remediation is feasible for MS patients.

In addition, we examined the utility of the SOP training intervention to improve speed of information processing as well as other cognitive skills that may be impacted by SOP including memory and executive function. First, we observed significant improvements on some measures of processing speed (Stroop, PASAT), although not others (SDMT). Our sample size was small and it is possible that improvements across all measures of processing speed would be found in a larger study. Second, significant improvements were seen on verbal memory measures (SRT), demonstrating that SOP training may impact other cognitive abilities. These findings are consistent with previous studies utilizing both SOP and working memory interventions, which showed improvement across several cognitive domains [29, 30]. However, to our knowledge no specific intervention exclusively targeting SOP has been conducted in MS. Although it is challenging to solely train processing speed given that other cognitive skills including divided attention are involved, processing speed was a primary component of the SOP intervention used in this study, and our findings suggest that SOP training may impact processing speed as well as other related cognitive functions. It is important to point out, however, that while improvements on some measures of processing speed and verbal memory were statistically significant, the observed changes were small and may not have had clinical significance. Also, we did not assess whether the observed changes were maintained after the completion of SOP training. It is not clear why no improvements were seen in visual learning and memory, which might have been expected given the visual nature of the SOP intervention.

We did not see significant changes in self-reported functional activities, emotional function, or HRQOL following the intervention, suggesting that improvements in cognitive measures had limited real-word impact. The fact that not all subjects demonstrated cognitive impairment at baseline could explain that lack of an effect on PRO outcomes. Ball et al. [23] concluded that the small effect size on functional outcomes seen in the ACTIVE trial was related to most subjects not being impaired in the domain of training. We also note that some subjects had fairly high subjective ratings of depression at baseline, which perhaps played a role in limiting any functional gains. Additionally, in order to restrict the intervention to tasks that have processing speed as their primary component, the intervention utilized only five tasks with 60% of the training time being devoted to one particular task. The repetitive nature of our intervention was a weakness of the study as some subjects reported boredom due to the lack of novel tasks. Perhaps if the tasks were perceived as more enjoyable, subjective mood and quality of life measures may have improved after the intervention. Further, the role of perceived cognitive function was not examined in this study. It is possible that subjects who perceived greater cognitive benefit from the intervention may have reported greater changes in functional activities, emotional function, and HRQOL. Future studies should include a measure of perceived cognitive function.

Participation in this study was not limited to individuals with documented deficits in speed of information processing. While it is possible that greater improvements in cognitive function and HRQOL would have been found by selecting subjects with impairment at baseline, our study may have broader implications for MS patients with varying levels of cognitive function. This is important as both healthy individuals and those with neurologic diseases are increasingly seeking out interventions to potentially delay the onset of cognitive decline. Several online cognitive brain training platforms including Posit Science's Brain HQ specifically cite the use of programs to promote brain plasticity for a range of individuals. Further, several studies in MS have shown that those with less severe baseline cognitive deficits may benefit from cognitive interventions the most [41]. Additionally, a recent study found that baseline cognitive impairment was not predictive of change in overall cognitive function or in response to intervention [32]. These findings provide support for the role of cognitive interventions in individuals with MS, regardless of level of cognitive function. Such interventions have implications for promoting cognitive reserve, which may delay or prevent the onset of cognitive difficulties.

A limitation of this study is that it did not include a control group to account for nonspecific treatment effects such as interactions with research staff and computer time. In addition, the lack of a control group made it difficult to determine whether the observed improvements on cognitive measures were due to practice effects. While alternate forms were used on verbal and visual memory measures, the improvements on SOP measures may have been due to task familiarity. A larger follow-up study should incorporate a control group to better assess the potential impact of practice effects. A different approach to reduce practice effects would be to conduct repeated cognitive assessments prior to starting the intervention, although one of the main goals of the study was to determine whether at-home training was feasible for MS patients and thus we sought to limit the number of study visits required.

Another limitation is that our subjects generally had minimal disability as measured by the Expanded Disability Status Scale, were primarily those with relapsing-remitting MS, and had minimal cognitive impairment at baseline. The majority were also employed at baseline and follow-up, reflecting a relatively high functioning group. It is thus unclear whether this intervention is feasible in those with greater physical and cognitive disability. Future studies should include more individuals with progressive disease subtype. This is important as patients with greater disability may have difficulty attending in-person sessions and therefore may benefit significantly from at-home cognitive training.

A final study limitation is related to the SOP training intervention itself. While processing speed is a primary component of the intervention, most tasks of processing speed require and affect multiple cognitive and sensory functions simultaneously [42]. We attempted to train one specific cognitive skill in contrast to other studies which have targeted multiple cognitive abilities, thus limiting the interpretation of any task-specific treatment effects. Although our intervention largely targeted processing speed, possible effects of training on other cognitive skills such as attention cannot be easily parceled out. Future studies may benefit from more specific training aimed at targeting processing speed exclusively.

In summary, this study supports the feasibility of an at-home SOP training intervention for individuals with MS. Our findings, in conjunction with findings from other recent studies, have important clinical implications for individuals with MS who are concerned about improving or preserving cognitive function. We found some evidence of improvement despite a minimal time commitment (1.5-2 hours weekly), which may be feasible for a range of individuals with MS including those who continue to work full time and/or have family and other commitments. Neuropsychologists, neurologists, speech therapists, occupational therapists, and neurophysiotherapists caring for MS patients should begin to consider alternative or complimentary forms of cognitive remediation including at-home training as traditional cognitive rehabilitation is not appropriate for all types of patients due to accessibility issues, cost, and the time commitment required.

A larger follow-up to this pilot study would benefit from the addition of a control group, the inclusion of individuals with diverse disease subtypes and disability levels, and the administration of measures of perceived cognitive function. The use of additional tasks of processing speed for the SOP training intervention should also be considered, as well as more rigorous screening for visual impairments. The inclusion of a study trainer to offer encouragement and task-specific strategies might improve both subject performance and compliance. Finally, follow-up testing months to years after the completion of the intervention should be incorporated to determine if training effects are maintained.

## Figures and Tables

**Table 1:** Demographic characteristics of study subjects.

N	15
Age (mean (SD); years)	47.0 (6.3)

Female (N (%))	12 (80.0)

White (N (%))	12 (80.0)

Disease duration (mean (SD); years)	14.4 (5.6)

EDSS (median (range))	1.75 (0, 6)

Disease category (RR/SP)	14/1

*Note. *EDSS = Expanded Disability Status Scale

**Table 2:** Summary statistics for cognitive measures.

	Baseline	Post-intervention	Change Including All Subjects	Change Only in Completers
	(Mean+/-SD)	(Mean+/-SD)	(Estimate; 95% CI; p-value)	(Estimate; 95% CI; p-value)
*Cognitive measures*				

SRT LTS	44.13+/-16.59	55.75+/-10.75	9.45; 95% CI: (2.85,16.06); p=0.009	8.75; 95% CI: (2.21,15.29); p=0.013

SRT CLTR	33.67+/-15.25	45.42+/-15.97	10.43; 95% CI: (3.38,17.48); p=0.008	10; 95% CI: (2.96,17.04); p=0.01

SRT Intrusions	1.27+/-1.53	1.67+/-2.35	0.41; 95% CI: (-0.74,1.56); p=0.449	0.42; 95% CI: (-0.78,1.61); p=0.459

SRT Delayed Recall	7.87+/-3.25	9.33+/-2.02	1.26; 95% CI: (-0.12,2.64); p=0.071	1.17; 95% CI: (-0.21,2.54); p=0.089

10/36 Total Correct	23.67+/-4.2	23.83+/-3.1	0.05; 95% CI: (-2.85,2.96); p=0.968	-0.42; 95% CI: (-3.5,2.67); p=0.772

10/36 Delayed Recall	8.27+/-1.94	8.33+/-1.97	0.15; 95% CI: (-1.07,1.38); p=0.787	0.25; 95% CI: (-1.02,1.52); p=0.674

SDMT	62+/-18.31	64.08+/-17.52	2.32; 95% CI: (-1.47,6.11); p=0.205	2.33; 95% CI: (-1.46,6.13); p=0.203

PASAT 3	52+/-8.64	53.25+/-8.24	1.97; 95% CI: (-0.68,4.63); p=0.13	2.08; 95% CI: (-0.6,4.77); p=0.116

PASAT 2	38.93+/-10.44	43.58+/-10.92	5.04; 95% CI: (1.92,8.15); p=0.004	5.08; 95% CI: (1.95,8.21); p=0.004

Stroop Word	99.27+/-9.07	103+/-11.6	3.95; 95% CI: (0.19,7.72); p=0.041	4; 95% CI: (0.18,7.82); p=0.042

Stroop Color	76.53+/-8.79	76.67+/-10.33	2.03; 95% CI: (-0.21,4.27); p=0.072	2.17; 95% CI: (-0.08,4.41); p=0.057

Stroop Color-Word	43.2+/-6.35	45.5+/-8.12	3.45; 95% CI: (-0.23,7.12); p=0.063	4; 95% CI: (0.32,7.68); p=0.036

COWAT	34.4+/-10.71	39+/-8.8	3.19; 95% CI: (-2.57,8.95); p=0.248	2.17; 95% CI: (-3.56,7.89); p=0.423

SRT LTS = Selective Reminding Test Long Term Storage; SRT CLTR = Selective Reminding Test Consistent Long Term Retrieval; SRT Intrusions = Selective Reminding Test Intrusions; SRT Delayed Recall = Selective Reminding Test Delayed Recall; 10/36 Total Correct = 10/36 Spatial Recall Test Total Correct; 10/36 Delayed Recall = 10/36 Spatial Recall Test Delayed Recall; SDMT = Symbol Digit Modalities Test; PASAT 3 = Paced Auditory Serial Addition Test 3 Second Trial; PASAT 2 = Paced Auditory Serial Addition Test 2 Second Trial; Stroop W = Stroop Word Reading Trial; Stroop C = Stroop Color Trial; Stroop Color Word = Stroop Color-Word Interference Trial; COWAT = Controlled Oral Word Association Test.

**Table 3:** Summary statistics for patient reported outcome measures.

	Baseline (Mean+/-SD)	Post-intervention (Mean+/-SD)	Change Including All Subjects	Change Only in Completers
			(Estimate; 95% CI; p-value)	(Estimate; 95% CI; p-value)
*Patient reported outcome measures*				

SF-36 Physical functioning	45.68+/-10.76	46.75+/-8.35	-1.12; 95% CI: (-3.44,1.2); p=0.312	-1.25; 95% CI: (-3.57,1.06); p=0.258

SF-36 Role physical	40.28+/-13.83	39.54+/-13.32	-0.63; 95% CI: (-4.33,3.08); p=0.716	-0.62; 95% CI: (-4.34,3.11); p=0.723

SF-36 Bodily pain	45.21+/-10.62	52.37+/-8.9	5.96; 95% CI: (-0.15,12.06); p=0.055	4.88; 95% CI: (-1.07,10.83); p=0.098

SF-36 General health	42.46+/-12.27	45.72+/-12.87	1.84; 95% CI: (-1.05,4.73); p=0.189	1.74; 95% CI: (-1.17,4.64); p=0.215

SF-36 Vitality	42.31+/-12.77	44.1+/-9.54	0.72; 95% CI: (-2.65,4.1); p=0.646	0.6; 95% CI: (-2.78,3.98); p=0.704

SF-36 Social functioning	46.9+/-12.29	47.09+/-10.34	0.8; 95% CI: (-3.39,5); p=0.681	0.93; 95% CI: (-3.3,5.17); p=0.638

SF-36 Role emotional	45.57+/-10.79	45.57+/-12.91	-0.53; 95% CI: (-6.77,5.7); p=0.854	-0.84; 95% CI: (-7.23,5.55); p=0.777

SF-36 Mental health	51.68+/-9	49.9+/-9.53	-1.52; 95% CI: (-4.32,1.29); p=0.259	-1.48; 95% CI: (-4.32,1.36); p=0.276

Sf-36 Physical composite summary	41.63+/-11.66	45.36+/-8.44	2; 95% CI: (-1.56,5.55); p=0.242	1.73; 95% CI: (-1.8,5.27); p=0.304

SF-36 Mental composite summary	49.03+/-8.76	47.73+/-10.75	-0.9; 95% CI: (-4.1,2.31); p=0.549	-0.83; 95% CI: (-4.09,2.42); p=0.585

CES-D Total	29.73+/-8.32	29.75+/-8.77	0.41; 95% CI: (-3.88,4.69); p=0.838	0.58; 95% CI: (-3.71,4.88); p=0.771

WPAI Activity Impairment	21.33+/-22	32.08+/-31	11.06; 95% CI: (-3.26,25.38); p=0.117	11.25; 95% CI: (-3.35,25.85); p=0.118

SF-36 Physical functioning = Medical Outcomes Study Short Form 36 Health Survey Physical functioning; SF-36 Role physical = Medical Outcomes Study Short Form 36 Health Survey Role physical; SF-36 Bodily pain = Medical Outcomes Study Short Form 36 Health Survey Bodily pain; SF-36 General health = Medical Outcomes Study Short Form 36 Health Survey General health; SF-36 Vitality = Medical Outcomes Study Short Form 36 Health Survey Vitality; SF-36 Social functioning = Medical Outcomes Study Short Form 36 Health Survey Social functioning; SF-36 Role emotional = Medical Outcomes Study Short Form 36 Health Survey Role emotional; SF-36 Mental health = Medical Outcomes Study Short Form 36 Health Survey Mental health; SF-36 Physical composite summary = Medical Outcomes Study Short Form 36 Health Survey Physical composite summary; SF-36 Mental composite summary = Medical Outcomes Study Short Form 36 Health Survey Mental composite summary; CES-D Total = Center for Epidemiological Studies Depression Scale Total; WPAI Activity Impairment = Work Productivity and Activity Impairment Questionnaire Activity Impairment.

## Data Availability

The data used to support the findings of this study are available from the corresponding author upon request.
